# The capture proteasome assay (CAPA) to evaluate subtype-specific proteasome inhibitors

**DOI:** 10.1016/j.dib.2015.04.019

**Published:** 2015-05-18

**Authors:** Nathalie Vigneron, Joanna Abi Habib, Benoît J. Van den Eynde

**Affiliations:** a Ludwig Institute for Cancer Research, Avenue Hippocrate 74, UCL 7459, B-1200 Brussels, Belgium; b WELBIO (Walloon Excellence in Life Sciences and Biotechnology), Brussels, Belgium; c de Duve Institute, Université catholique de Louvain, Brussels, Belgium

**Keywords:** Proteasome, Activity assay, Fluorogenic peptides, Proteasome-Glo

## Abstract

We recently developed a new assay to measure proteasome activity *in vitro* (CAPA for capture proteasome assay) [1], based on proteasome capture on an antibody-coated plate. When used with lysates originating from cells expressing either standard proteasome, immunoproteasome or intermediate proteasomes β5i or β1i-β5i, this assay allows the individual monitoring of the chymotrypsin-like, trypsin-like and caspase-like activities of the corresponding proteasome subtypes. The efficiency and specificity of four proteasome inhibitors were studied using the CAPA assay, demonstrating the potential of this assay for the development of subtype-specific proteasome inhibitors.

Specifications Table

**Table:** 

Subject area	*Biology, Biochemistry*
More specific subject area	*Proteasome and its function*
Type of data	*Figures and table*
How data was acquired	*Fluorescence microplate reader*
Data format	*Analyzed*
Experimental factors	*Not applicable*
Experimental features	*Proteasomes are captured on a microplate and fluorogenic peptides are used to measure proteasome activity in vitro*
Data source location	*Not applicable*
Data accessibility	*Not applicable*

**Value of the data**

• Measure of proteasome activity based on a single step of proteasome antibody capture.
• Bypasses the heavy and complex procedures of proteasome purification.
• Eliminates the need of using protease inhibitors for result interpretation.
• Useful for the identification of proteasome subtype-specific inhibitors.
• Assay performed in the absence of MgSO_4_, which affects proteasome function.

## 1 Data, experimental design, materials and methods

### 1.1 Data

**Fig. 1:**
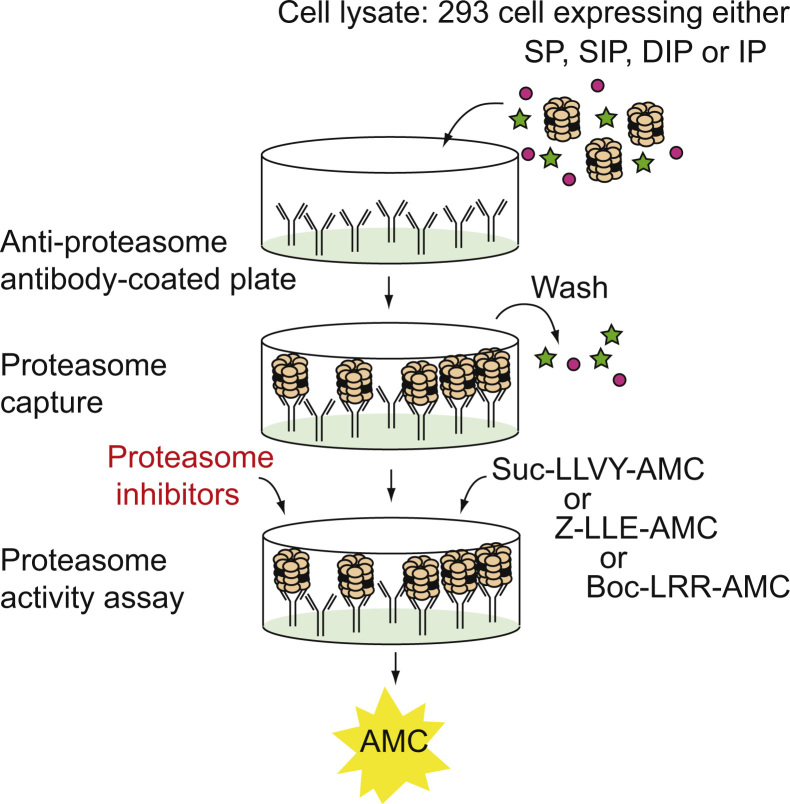
Representation of the CAPA assay. Lysates from 293 cells expressing standard proteasome (SP), immunoproteasome (IP) or intermediate proteasomes β5i (SIP) or β1i-β5i (DIP) were loaded on a 96-well black Maxisorp plate pre-coated with the anti-α2 antibody MCP21. Following proteasome capture, the plate is carefully washed and proteasome activity was measured using substrates Suc-LLVY-AMC, Z-LLE-AMC or Boc-LRR-AMC, which are specific for the chymotrypsin-like, caspase-like and trypsin-like activities, respectively.

**Fig. 2:**
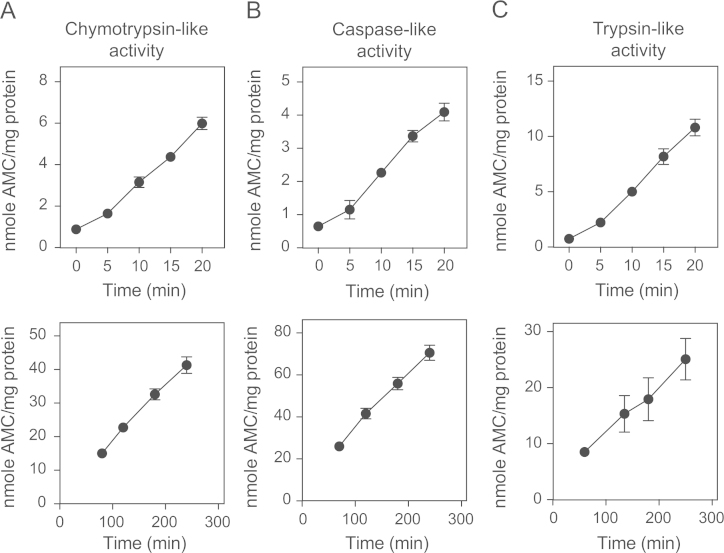
Kinetics of AMC release during the CAPA assay over time. Lysates from 293 cells expressing immunoproteasome (A,C) or standard proteasome (B) were loaded on a 96-well black Maxisorp plate pre-coated with the anti-α2 MCP21 antibody. Following proteasome capture, the plate was carefully washed and proteasome activity was measured using the substrates Suc-LLVY-AMC, Z-LLE-AMC or Boc-LRR-AMC, which are specific for the chymotrypsin-like, caspase-like and trypsin-like activities, respectively. After addition of the fluorogenic substrates, AMC release (±SD) was recorded over time at 37 °C either for short time intervals (upper graphs) or longer time intervals (lower graphs).

**Fig. 3:**
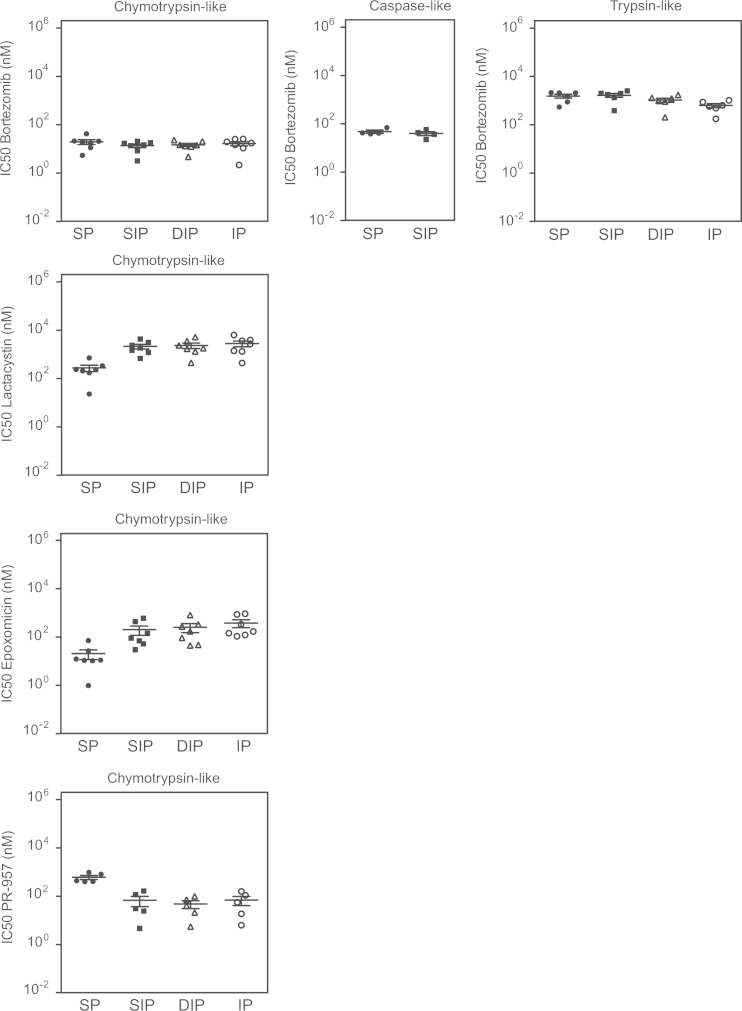
Reproducibility of IC50 determination for the four proteasome inhibitors. Lysates from 293 cells expressing standard proteasome (SP), immunoproteasome (IP) or intermediate proteasomes β5i (SIP) or β1i-β5i (DIP) were loaded on a 96-well black Maxisorp plate pre-coated with the anti-α2 antibody MCP21. Following proteasome capture, the plate was carefully washed and the proteasome inhibitor was added at different concentrations. Proteasome activity was then measured using the substrates Suc-LLVY-AMC, Z-LLE-AMC or Boc-LRR-AMC, which are specific for the chymotrypsin-like, caspase-like and trypsin-like activities, respectively. Each symbol represents the IC50 measured in one experiment performed in duplicates. Lines indicate means ± SEM.

To facilitate the *in vitro* study of proteasome activity, we have designed a new type of assay (CAPA for capture proteasome assay) based on the specific capture of proteasomes on 96-well plates [1] (Fig. 1). Using this assay, proteasomes contained in any type of human cell lysate are captured on a black Maxisorp plate pre-coated with the anti-proteasome α2 subunit antibody MCP21 and tested for their ability to degrade fluorogenic peptides suc-LLVY-AMC, Z-LLE-AMC or Boc-LRR-AMC. This enables the measurement of the chymotrypsin-like, caspase-like and trypsin-like activities of the proteasome, respectively (Fig. 1). Applying the CAPA assay to lysates from cells expressing either standard proteasome (SP), immunoproteasome (IP) or intermediate proteasomes β5i (SIP) or β1i-β5i (DIP) [2], we could monitor the activity of each proteasome subtype [1]. As shown in Fig. 2, fluorescence emission follows a linear slope over time, thereby enabling the quantitative measurement of proteasome activity up to at least four hours after addition of the substrate. The development of subtype-specific proteasome inhibitors is the focus of intensive investigations in the field of anti-cancer therapy as well as inflammation-related autoimmune diseases. In that regard, the CAPA assay, coupled to the use of the 293-EBNA cell lines expressing either proteasome type represents a tool of choice to facilitate the study of proteasome subtype inhibitors. As a proof-of-concept, we have used the CAPA assay to test the effect of four different proteasome inhibitors (bortezomib, lactacystin, epoxomicin and PR-957) on the chymotrypsin-like, caspase-like and trypsin-like activities of the four proteasome subtypes (see Figure 7 and 8 in Ref [1]). The mean of the IC50 values measured for each inhibitor on a given proteasome subtype are reported in Table 1. To illustrate the robustness of the CAPA assay for measuring proteasome inhibition *in vitro*, Fig. 3 displays the different IC50 values measured in a series of independent experiments. Our results show that the dipeptide boronic acid bortezomib blocked the chymotrypsin-like activity and caspase-like activity with IC50 values of ~15 nM for the chymotrypsin-like activity and ~40 nM for the caspase-like activity (Figure 7A in Ref [1], Table 1 and Fig. 3). Bortezomib was effective at blocking the chymotrypsin-like activities of the four types of proteasomes, showing that it targets equally β5 or β5i. As expected, the concentration of lactacystin required to block the chymotrypsin-like activity of the proteasome was much lower than that necessary to block the caspase-like and trypsin-like activities (Figure 7B in Ref [1], Table 1 and Fig. 3). Interestingly, lactacystin inhibited more effectively the chymotrypsin-like activity of the SP, suggesting that it targets more efficiently the β5 subunit than the β5i subunit. Similar results were obtained with epoxomicin (Figure 8A in Ref [1], Table 1 and Fig. 3). Proteasome inhibitor PR-957, which is known to specifically target β5i, efficiently blocked the chymotrypsin-like activity of the IP, the SIP and the DIP, while being much less efficient at inhibiting the chymotrypsin-like activity of the SP (Figure 8B in Ref [1], Table 1 and Fig. 3). PR-957 was not effective on the caspase-like and trypsin-like activities of the proteasomes. Overall these results show the potential of the CAPA assay for the study and identification of proteasome subtype-specific inhibitors.

### 1.2 Experimental design and methods

#### 1.2.1 Cell lines

293 cells expressing the standard proteasome (β1-β2-β5), the immunoproteasome (β1i-β2i-β5i), the intermediate proteasome β5i (SIP) or β1i-β5i (DIP) [2] were grown in Iscove׳s Modified Dulbecco׳s Medium (IMDM, Thermo Scientific Inc., Waltham, MA, USA) containing 10% fetal calf serum (Thermo Scientific) and supplemented with Puromycin (5 µg/ml, Sigma, St Louis, MA, USA) and/or Hygromycin (600 µg/ml, InvivoGen, San Diego, CA, USA). All culture media were supplemented with L-arginine (116 mg/l), L-asparagine (36 mg/l), L-glutamine (216 mg/l), penicillin (100 U/ml) and streptomycin (100 mg/ml) (Thermo Scientific). The MCP21 hybridoma was obtained from ECACC and the antibody was purified from hybridoma supernatant using HiTrap Columns Prepacked with Protein G Sepharose [3].

#### 1.2.2 Capture proteasome assay (CAPA)

Black 96-well maxisorp plates (VWR, Radnor, PA, USA) were coated using 5 µg/ml MCP21 antibody and then further blocked for 1 h in PBS containing 2% BSA. Cell pellets were washed in PBS and lysed on ice at a cell density of 10^7^ cells/ml in TRIS 50 mM NP40 0.1% pH 7.5. Post nuclear supernatant is then collected, analyzed using the BCA protein assay kit (Thermo Scientific) and adjusted to a concentration of 200 µg per ml in lysis buffer (Tris 50 mM NP40 0.1% pH 7.5). 50 µl of cell lysate is then added in each well and the plates were incubated for 2 h at 4 °C. The amount of proteasome captured in these conditions was estimated to be around 200 ng per well using quantitative ELISA [2]. After proteasome capture, plates were carefully washed in Tris 20 mM NP40 0.1% pH 7.5 then in Tris 20 mM pH 7.5 to remove traces of detergent. Proteasome inhibitors diluted in Tris 20 mM pH 7.5 were then added to the wells and incubated under rotation for 10 min at room temperature. Finally, fluorogenic substrates Suc-LLVY-AMC, Z-LLE-AMC or Boc-LRR-AMC (Enzo, Farmingdale, NY, USA) were added to the wells at a concentration of 100 µM in Tris 20 mM. Plates were then sealed with a plastic cover and incubated at 37 °C for about 30 min. Fluorescence emission was then measured using the proteasome-GloMAX apparatus (Promega, Fitchburg, WS, USA) or the SpectraMax 190 Microplate Reader (Excitation 380 nm, Emission 460 nm). The linearity of fluorescence emission over time is verified by performing measurements at different time points as shown in Fig. 2. The IC50 values were calculated in Prism 5.0 (Graphpad, San Diego, CA, USA) from a log (inhibitor) vs response curve (variable slope) using the equation *Y*=Bottom+(Top-Bottom)/(1+10^((Log IC50-*X*)⁎HillSlope))^ where *X* is the logarithm of the inhibitor concentration and *Y* the proteasome activity.

## Figures and Tables

**Table 1:** IC50 of the four proteasome inhibitors, measured in the CAPA assay. Statistical significance of the difference between the IC50 measured for each proteasome subtype and the IC50 measured for the standard proteasome is indicated in upper case. ANOVA with Bonferroni׳s multiple comparison test.

	Inhibitor	Standard Proteasome IC50 (±SEM) nM	Intermediate Proteasome β5i IC50 (±SEM) nM	Intermediate Proteasome β1i β5i IC50 (±SEM) nM	Immunoproteasome IC50 (±SEM) nM
*Chymotrypsin-like activity*					
	Bortezomib (*n*=7)	20 (±4)	14 (±2)^ns^	15 (±2)^ns^	17 (±3)^ns^
	Lactacystin (*n*=7)	279 (±82)	2165 (±476)⁎⁎	2357 (±599)⁎⁎	2841 (±765)⁎⁎⁎
	Epoxomicin (*n*=7)	21 (±9)	207 (±86)^ns^	260 (±105) ^ns^	388 (±138)⁎⁎
	PR-957 (*n*=5)	618 (±121)	69 (±31)⁎⁎⁎⁎	48 (±17)⁎⁎⁎⁎	70 (±29)⁎⁎⁎⁎
*Caspase-like activity*					
	Bortezomib (*n*=4)	48 (±7)	41 (±7)^ns^	n.d.	n.d.
	Lactacystin (*n*=3)	>5000	>5000	n.d.	n.d.
	Epoxomicin (*n*=3)	>1000	>1000	n.d.	n.d.
	PR-957 (*n*=3)	>5000	>5000	n.d.	n.d.
*Trypsin-like activity*					
	Bortezomib (*n*=6)	1605 (±291)	1729 (±313)	1095 (±214)	653 (±127)
	Lactacystin (*n*=3)	>5000	>5000	>5000	>5000
	Epoxomicin (*n*=4)	>1000	>1000	>1000	>1000
	PR-957 (*n*=4)	>5000	>5000	>5000	>5000

ns: not significant.

⁎⁎ *p*≤0.01.

⁎⁎⁎ *p*≤0.001.

⁎⁎⁎⁎ *p*≤0.0001.
